# Lightweight GPS-Tags, One Giant Leap for Wildlife Tracking? An Assessment Approach

**DOI:** 10.1371/journal.pone.0028225

**Published:** 2011-12-07

**Authors:** Mariano R. Recio, Renaud Mathieu, Paul Denys, Pascal Sirguey, Philip J. Seddon

**Affiliations:** 1 School of Surveying, University of Otago, Dunedin, New Zealand; 2 Department of Zoology, University of Otago, Dunedin, New Zealand; 3 Earth Observation Research Group, CSIR-Natural Resource Environment, Pretoria, South Africa; Monash University, Australia

## Abstract

Recent technological improvements have made possible the development of lightweight GPS-tagging devices suitable to track medium-to-small sized animals. However, current inferences concerning GPS performance are based on heavier designs, suitable only for large mammals. Lightweight GPS-units are deployed close to the ground, on species selecting micro-topographical features and with different behavioural patterns in comparison to larger mammal species. We assessed the effects of vegetation, topography, motion, and behaviour on the fix success rate for lightweight GPS-collar across a range of natural environments, and at the scale of perception of feral cats (*Felis catus*). Units deployed at 20 cm above the ground in sites of varied vegetation and topography showed that trees (native forest) and shrub cover had the largest influence on fix success rate (89% on average); whereas tree cover, sky availability, number of satellites and horizontal dilution of position (HDOP) were the main variables affecting location error (±39.5 m and ±27.6 m before and after filtering outlier fixes). Tests on HDOP or number of satellites-based screening methods to remove inaccurate locations achieved only a small reduction of error and discarded many accurate locations. Mobility tests were used to simulate cats' motion, revealing a slightly lower performance as compared to the fixed sites. GPS-collars deployed on 43 cats showed no difference in fix success rate by sex or season. Overall, fix success rate and location error values were within the range of previous tests carried out with collars designed for larger species. Lightweight GPS-tags are a suitable method to track medium to small size species, hence increasing the range of opportunities for spatial ecology research. However, the effects of vegetation, topography and behaviour on location error and fix success rate need to be evaluated prior to deployment, for the particular study species and their habitats.

## Introduction

Global Positioning Systems (GPS) technology has progressively demonstrated its usefulness in wildlife tracking since the mid-nineteen nineties. Its extension into wildlife research has expanded the possibilities for studying the spatial ecology of animals to the extent that: “this powerful synergy between science and technology is rapidly shaping the discipline of ecology” [1]. Previous methods for collecting animal positions have mainly relied on traditional VHF-radiotracking and entailed a number of challenges that have largely been minimized or overcome by GPS technology. GPS tracking allows the collection of animal positions at higher rates and shorter intervals, in remote and poorly accessible areas, during all time and all weather conditions, and avoids modified animal behaviour due to the proximity of the researcher. In addition, GPS positional data is typically of greater accuracy than the ±70–600 m obtained from triangulation of VHF radio-signals [2], [3], and more accurate than the spatial detail provided in most available habitat maps [4]–[6]. Hence, wildlife GPS-tracking is considered as a suitable technique for investigations at fine ecological scales (10–250 m) [7].

Due to weight and size limitations, GPS devices have until recently been suitable only for tracking animals capable of carrying a relatively large receiver, thus limiting the range of species that can be tagged. However, some small GPS devices are starting to be used to track birds such as pigeons (*Columba livia*) [8], capercaillie (*Tetrao urogallus*) [9] and turkeys (*Meleagris gallapavo)* [10]. Improvements in microelectronics and battery technology now make it also possible to use GPS devices to track mammals of medium to small size for extended periods, hence, increasing the range of species that can be tracked [1], e.g., Japanese macaque (*Macaca fuscata*) [11], ocelots (*Leopardus pardalis*) [12], brushtail possums (*Trichosurus vulpecula*) [13], [14], feral and domestic cats (*Felis catus*) [15], [16], [17], and hedgehogs (*Erinaceous europaeus*) [18].

In spite of the many advantages and recent improvements in GPS tracking, factors affecting receiver performance must be considered. The two key measures used to quantify the probability of recording a position and its geolocation quality are: 1) fix success rate (FSR) or the proportion of successful fixes (i.e., successful location acquisition) per fix trial, and 2) location error (LE), or the linear distance between a fix position and a true reference position. FSR and LE depend on technological, environmental and behavioural factors that can affect the appropriate signal reception in space and in time.

The technological factors are varied; for example, the number of satellites used to compute a position and their associated geometric configuration in the sky are of prime importance [19]. At least three satellites are required in order to acquire a 2-dimensional (2-D) fix and a minimum of four satellites are needed for a 3-dimensional (3-D) fix [20]. Theoretically, the greater the number of satellite signals tracked and used to compute a position, the higher the accuracy. A theoretical estimation of the likely precision of a location fix, based solely on the satellite geometry, is expressed as the Dilution of Precision (DOP) (e.g., horizontal or HDOP, vertical or VDOP or 3D position or PDOP), whereby higher DOP values are associated with poor satellite constellation geometry [19]. DOPs are an indication of the possible accuracy of a position fix, and are frequently used in wildlife GPS-based projects for filtering locations [21], [22], where it is assumed that a high DOP indicates a high LE (i.e., low accuracy). A high DOP does not mean that the position fix is of low quality or positional accuracy, but rather that current satellite constellation could result in a low accuracy position fix. Additional factors affecting the LE and FSR include the satellite elevation in the sky, satellite clock accuracy, multipath signals related to reflected radio signal on near-by surfaces, ionospheric and tropospheric effects (atmospheric noise) that delay satellite signals, electronic malfunction, varied performance of receiver electronics between brands, and nulls while batteries fade [5], [19], [23].

The main non-technical factors affecting LE and FSR include vegetation composition and density, topography, and animal behaviour [3]. The last implies variations in signal reception due to body obstruction and changes in antenna position; these have been identified as a source of error for large terrestrial mammals while bedding, feeding or moving [6], [20], [21], [24], [25]–[28].

The level of error associated with vegetation and topography can be theoretically estimated with adequate information and analysis [25]. Hence, the best practice for any proposed wildlife project based on GPS tracking involves assessment of the receiver's errors [1], and understanding the magnitude and causes of variation in FSR and LE in the target study area. An assessment should consider the effect of environmental variables, the species being tracked, and the nature of the ecological questions being addressed. Previous GPS performance assessments have been carried out through a combination of stationary and animal behaviour tests using GPS embedded in collars. However, these tests focused on large terrestrial mammals, usually ungulates and carnivores inhabiting specific environments such as the boreal forest and habitats of North America and Canada (see [3]). As a result, currently summarized LE in wildlife GPS tracking accuracy is considered to be ±30 m, although locations with abnormally large LE values of up to several kilometers can occur intermittently for all GPS devices [3]. A review of 35 journal articles [29] revealed an average FSR of 94.8% in stationary tests and 69.3% in the case of GPS deployed on animals. However, performance appears to vary substantially among species and projects. For instance, FSR ranges from 49.4% [26] to 72% for grizzly bears (*Ursus arctos*) [6], 59%–95% for grizzly and black bears (*Ursus americanus*) [30], 69% [31] to 97% for elk (*Cervus Canadensis*) [23], 45%–85% for cougars (*Puma concolor*) [32], and 60% −70% for moose (*Alces alces*) [20].

The miniaturization of the electronic components and antenna of lightweight GPS-tags, as well as the size and behavioural patterns of targeted species are factors that may result in poorer performance in comparison with previously reported assessments. The proximity of the GPS device to the ground and the behaviour of small species (e.g., use of cavities and other micro-topographical features) may have pronounced effects on device errors. In addition, differences in sexual and seasonal behavioural patterns (e.g., foraging, mating, denning, breeding) can result in the tendency of tracked individuals to select habitats prone to increased GPS errors (e.g., areas with vegetation and/or topography blocking satellite signals, use of cavities or covered refuges). Hence, the development and application of lightweight GPS-devices suitable for tagging medium to small mammals requires specific assessments.

Cargnelutti et al. [25] tested GPS-collars of ca. 300 g, suitable for tracking medium size mammals, at stationary sites in open and forested habitats without considering topographic influences, and assessed the mobility effects on collar performance under forest using a pet dog. However, the current information on performance of collars <200 g is based on data obtained through deployment on animals [11]–[14]. Whenever preliminary tests were carried out, they were limited to open-sky conditions (i.e., no topographic or vegetation influence) [14], or tested in one site with specific environmental characteristics [13], and following protocols inherited from tests based on larger GPS-collars (i.e., deployments 1 m over ground) [11]–[14]. Hence, additional studies are required to identify and quantify the factors affecting the performance of lightweight GPS-collars (i) in a wider range of environmental conditions (i.e., topography and vegetation), (ii) at heights close to the ground so as to better mimic realistic positions of medium-to-small mammals, (iii) considering the effect of lower vegetation structures, alone or in conjunction with higher ones such as shrubs or tree canopy (i.e., understorey).

We assessed the main environmental, technical and behavioural causes of error in lightweight GPS-collars suitable for tagging medium to small terrestrial mammals. Our objectives were: 1) to evaluate the consistency in the performance of same-brand lightweight GPS-collars; 2) to determine the influence of the number of satellites and geometry configuration (expressed as the Horizontal Dilution of Position or HDOP) as major technical factors, and to extend conclusions to the evaluation of the suitability of HDOP/number of satellites-screening criteria for filtering less accurate positions from tracking datasets; 3) to quantify the influence of vegetation and topography on FSR and LE, also incorporating the effect of satellite number and their geometry configuration for LE analysis; 4) to evaluate the effect of collar motion in reducing the efficiency of the GPS performance; 5) to identify differences in FSR during a real deployment of lightweight GPS-collars on feral cats, that may be attributable to differences in sexual and seasonal behavioural patterns.

## Methods

### Ethics Statement

This project was conducted under University of Otago Animal Ethics Approval 14/08.

We assessed the performance of lightweight GPS-collars suitable for tracking mammals over approximately 2.5 kg in body mass. The collar used in all tests comprises a 125 g GPS data-logger (Sirtrack, Havelock North, NZ, http://www.sirtrack.com) equipped with a 12-channel GPS receiver Trimble iQ. Collars of this brand have been previously used to track feral cats [17] and brushtail possums [11] in New Zealand. Data were recorded in a built-in memory and included date, time, longitude, latitude, number of satellites, and the HDOP. Receivers were not manufactured to acquire data for post-processing differential correction. We followed an integrated experimental design where collars were (i) placed at known locations, either at a geodetic survey point or fixed points in varied habitats, (ii) moved along simulated small mammal tracks, and (iii) deployed on wild animals in the field.

Fix success rate was calculated per collar deployment by dividing the number of successful fixes by the number of possible fixes. Location error per fix was calculated as the planimetric Euclidean distance between the GPS-measured location and the reference position. One way to express the average location error of a GPS device extracted from a population of data fixes is the root mean square. For clarity, we refer to the location error of a fix as LE, the location error calculated from *n* fixes using the root mean square (RMS) as LE_RMS_ (LE_RMS_ = [(LE_1_^2^+LE_2_^2^+…+LE_n_^2^)/*n*]^0.5^), and the arithmetic mean of the location error of *n* fixes as µLE. The median of several *n* fixes was also calculated for information and comparison with previous publications. We used R [33] and Statistica® 6 (StatSoft, Tulsa, USA) for all the statistical analysis and modelling. Geographical analyses were carried out using ArcGIS™9.3 (ESRI, Redlands, CA).

### Study Area

The research took place in natural habitats of New Zealand, in two regions of the South Island, the Mackenzie Basin (central) and the Catlins (south east). Both regions include representative New Zealand terrestrial ecosystems ranging from grasslands to forests. The Mackenzie Basin is a dryland area in an intermontane depression basin of tectonic origin [34] limited to the west by the Southern Alps and extending approximately 40 km to the east and about 100 km from north to south. It is characterized by extensive semi-arid plains, gentle hills and conspicuous mountains shaped by intense glacial activity with conformed ‘U’ shape valleys today occupied by braided rivers. Vegetation is dominated by tussock grasslands well distributed from the plains to high-altitude slopes, and shrublands of native and exotic species. Forests are scarce and patchy, dominated by proliferating exotic conifers, although native forests of mountain beech (*Nothofagus* spp.) are more extensively present in the upper regions of valleys and occasionally as small patches in the lower reaches.

The Catlins region is a rugged area dominated by ranges of hills and containing the largest native forests on the east coast of the South Island, comprising temperate rain forests of native species of trees such as rimu (*Dacrydium cupressinum*), tōtara (*Podocarpus totara*), Southern rātā (*Metrosideros umbellata*), Kāmahi (*Weinmannia racemosa*), silver beech (*Nothofagus menziesii*) and mature manuka (*Leptospermum scoparium*). Different species grow under the native forest canopy composing diverse understorey of fern trees (*Cyathea smithii*, *Dicksonia squarrosa*), young lancewoods (*Pseudopanax crassifolius*) and brackens (*Pteridium* spp.).

### Stationary assessment

#### Receiver fault and consistency check

To verify the consistency of Sirtrack lightweight GPS-collars and to assess manufacturing failures, we simultaneously deployed five collars at a selected survey mark, without topographical or vegetation obstructions (i.e., open sky). Fixes were taken every 15 min during 24 hours to cover two full GPS satellite constellation cycles (i.e., 97 possible fixes). We investigated FSR and LE_RMS_ differences between collars. Significant differences between collars in regards with LE values were tested using a one-way analysis of variance (ANOVA, [35]).

#### Number of Satellites and HDOP

We analyzed the general performance of lightweight GPS-collars in regards to satellite geometry configuration by relating LE and the corresponding values of HDOP for all sampled locations (survey mark, and field sites) using a linear model [36]. We graphically analyzed the tendency of LE to fluctuate with increases in HDOP values, by calculating µLE and the standard deviation of locations associated with a specific HDOP value to visualize data dispersion. We also characterized the effect of data-filtering using a DOP criterion, by virtually removing the locations with HDOP that exceeded an incremental filtering threshold from 7 up to 12. The 100%, 95% and 50% percentile of LE values per HDOP threshold was calculated selectively for the data retained and data removed in order to assess the effect of HDOP-based screening in LE. LE_RMS_ values calculated from retained locations per threshold were related with the percentage of removed locations to identify the most suitable trade-off for HDOP filtering that effectively reduces LE_RMS_ while retaining most recorded locations.

#### Environmental and technical factors

In order to identify and quantify the environmental (topography, vegetation) and technical (HDOP, number of satellites) factors affecting FSR and LE, stationary tests were conducted with all five collars. Stationary tests have been widely used to evaluate the performance of GPS collars under different environmental configurations through the deployment of collars on sticks simulating the height of large mammals [3]. This approach does not account for the effect of animal activity or behaviour, resulting in higher FSR estimations in comparison with live animal tracking [24]. However, stationary tests allow for controlled replicates and assessment of the effects of different terrain and vegetation configurations [24], and also reveal the measurement error distribution around a reference position that can assist in buffer size selection around locations or other correction techniques [3].

A total of 60 sites were selected across the two study areas (i.e., Mackenzie Basin and Catlins), covering a range of vegetation and topographic settings characteristic of New Zealand environments. Site selection considered varied configurations of vegetation within three categories of sky availability, defined as the amount of sky not blocked by topographic features at the selected site. Collars were placed on 20 cm high sticks, simulating the height of small mammals, e.g., feral cat, and programmed to collect positions every 15 min over a 24-hour period. A mapping-grade professional GPS Leica® GS20 of sub-meter accuracy was used to define the reference coordinates of each site. The reference position was calculated by differentially correcting and averaging the individual positions fixed every 10 s during a 15-minute period.

#### Topographic covariates

Sky obstruction by surrounding slopes potentially blocks the line of sight between GPS satellites and the GPS receiver. As fewer satellites become visible (e.g., in steep valleys), the availability and/or quality of GPS-derived location is compromised due to weak or insufficient configuration geometry. In order to assess this effect on our collars, a raster layer was prepared to quantify sky availability *V_d_* (Figure S1). *V_d_* is defined as the portion of the overlying hemisphere that is visible from a point in the landscape. This is equivalent to the ratio between the solid angle subtended by the horizon lines and 2π [37]. It ranges from 0 for a totally obscured sky to 1 for an unobstructed horizontal surface. Dozier et al. [38] described an efficient horizon algorithm, based on the analysis of a digital elevation model (DEM) to determine the horizon angle *H(* 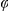 *)* in all azimuth angles 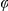. The sky visibility is computed as:
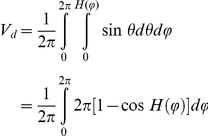
The sky availability layer was produced at a spatial resolution of 125 m based on a DEM interpolated from topographic contour lines. We considered three classes of sky availability: ‘Low sky availability’ (0.41–0.75), ‘Medium sky availability’ (0.78–0.84) and ‘High sky availability’ (0.87–1.00). In addition, we estimated aspect and slope on-site using a compass and a clinometer, respectively.

#### Vegetation covariates

Within the ‘high sky availability’ category, we selected nine classes of dominant vegetation types: ‘no vegetation’, ‘low vegetation’ (i.e., herbs, low ferns, tall grass), ‘medium density tussockland’ (50–75%), ‘high density tussockland’ (>80%), ‘medium density shrubland’ (50–75% shrub cover), ‘high density shrubland’ (>80%), ‘mature pine forest’, ‘native forest’, and ‘native forest with understorey’. The category ‘no vegetation’ acts as control with no vegetation above the collar height (i.e., >20 cm, e.g., bare ground, gravel surface, short grass). Forest understorey is characterized by large leaved plants to capture the scarce light penetrating the tree canopy. Hence, we evaluated the additional effect of this vegetation layer on FSR and LE. For ‘low vegetation’ and ‘forest’ habitat categories, sites were selected based on having at least 70% vegetative cover. In the case of the ‘medium density tussockland’ and the ‘medium density shrubland’ categories, the collar was placed both in gaps between tussock or shrub clumps, and also underneath clumps. We aimed to characterize an overall (or average) GPS performance to explain the variability of errors in highly heterogeneous habitats as perceived at the height of an animal of cat size (i.e., 20 cm). For the ‘medium’ and ‘low sky availability’ categories, we tested the most ‘extreme’ conditions affecting FSR and LE, thus we limited the tests to ‘high density tussockland’, ‘high density shrubland’ and ‘native forest’ habitats. The combination of topographic and vegetation categories produced 20 environmental settings. We used three similar sites (replicates) for each of these configurations separated at least by 500 m and placed at more than 50 m from the edge of the vegetation patch.

A set of quantitative vegetation variables was collected at each site in addition to the category of habitat type. We quantified canopy measurements using two approaches [39]: (i) canopy closure, measured as the proportion of vegetation covering the sky hemisphere from one view point (i.e., projection of hemisphere onto a plane), and (ii) canopy cover measured as the proportion of ground covered by the vertical projection of vegetation crowns. Hemispherical photography was used to quantify canopy closure [40]. Photographs were taken with a digital camera equipped with a fisheye lens (approx. 180° open angle), placed at the collar location, levelled and pointing upward toward the canopy. We analyzed every canopy closure photograph applying a contrast technique to determine binary pixels values of 0 (sky) and 1 (vegetation) using Adobe Photoshop 8.0.1.; thus canopy closure was defined as the percentage of sky blocked by the canopy divided by the total number of pixels in the image. The canopy cover of trees, shrubs, tussocks and low vegetation (grass, herbs, small ferns), respectively, was visually estimated around a buffer circle of 25 m radius from the collar position. In addition, we assessed the height of trees and shrubs contained in the 25 m buffer by averaging five measurements of random trees/shrubs using a clinometer. Average “diameter at breast height” DBH was also measured for the same five random trees.

#### GPS performance assessment and modeling

The FSR was computed for each site. LE was calculated per successful location and LE_RMS_ within each vegetation and topographic category and globally for all sites. We analyzed the presence of abnormal LE values (i.e., outliers) that intermittently occur in all GPS devices [3], and excluded them from model development by retaining data that fell within three times the standard deviation of the mean (µLE) for each site.

We used a model selection approach with the aim of identifying the predictor variables in relation to vegetation and terrain that have the greatest impact on FSR and LE separately. For both analyses, we first screened against collinear numeric covariates using Spearman pairwise correlation coefficient (|r|>0.7, cut-off value [41]) to avoid correlated variables within the same model. The influence of vegetation and terrain on FSR was assessed using fixed effect logistic regression [36] to model the probability of successful locations per site (i.e., expressed as the percentage of successful locations over the 97 possible) [5]. We fitted 14 different models representing the alternative logistic hypothesis plus the global and null model. Predictor variables included in the model process were: ‘habitat type’, ‘canopy closure’, ‘sky availability’, ‘slope’, ‘aspect’, cover of low vegetation (grass, herbs, small ferns), tussocks, shrubs and trees, as well as ‘tree height’ and ‘tree diameter’.

In contrast to FSR modelling, whereby technical variables (i.e., number of satellites and HDOP) are not available for missing locations, LE was modelled considering the same environmental covariates but adding the information collected in each fix for the ‘number of satellites’ and the associated HDOP values. Hence, the dependant variable included all LE values of fixes for the 60 sites up to 97 fixes per site. We used Linear Mixed Models (LMMs) and a random intercept added for each site to control for non independence of the fixes collected [23], [42]. We compiled 28 models as alternative hypothesis plus a constant null model.

For both analyses, we compared a set of models representing a priori hypotheses of variables affecting LE and FSR. We used the Akaike Information Criterion (AIC) for LE, and AIC adjusted for small sample sizes and dispersed data (*Q*AIC_c_) for FSR. We model-averaged the coefficients for the predictors in each model set [43].

### Motion and behavioural assessment

To complement the information derived from stationary positions, we tested collar performance (FSR and LE_RMS_) by simulating cat motion using one collar with an acquisition rate of one fix per minute. To simulate motion comparable to that of a cat-size mammal we designed a sled-like device to hold the collar at 20 cm above ground, made out of 3 mm mouldable but rigid wire shaped at one end to hold the collar, pivoting over one point on the ground, and easily sliding over any surface without snagging (Figure S2). The device was pulled over the ground maintaining a two meter distance between the operator and collar to reduce any blocking-effect of the GPS signal caused by the operator's body. The operator walked haphazardly along routes going through mosaics of representative landscapes of the Mackenzie Basin of New Zealand (grassland, tussockland, and shrubland), at different times of the day and in areas of high sky visibility. Five different routes and simulated movements of varied length, velocity or sinuosity were used to approximate those of a cat's track. Reference itineraries were recorded simultaneously using a Garmin ® Map 60CSx GPS (RMS accuracy = ±4.5 m tested during 9 different hours) which was held two meters above the ground collecting positions at intervals of approximately 1–2 seconds. The Garmin position collected within 2 seconds of a collar location was considered as the reference position. LE was calculated as the Euclidean distance between the two corresponding spatial coordinates collected at the same time.

To assess differences in FSR resulting from behavioural patterns of animals associated with gender or season, we analyzed the positional data derived from 14 Sirtrack GPS-collars deployed on 43 feral cats in the Tasman [17] and Godley valleys (Mackenzie Basin area). For instance, male and female feral cats may exhibit variation in denning behaviour and seasonal selection of habitats prone to decrease FSR. A total of 24 males and 19 females were collared for ca. 2 weeks covering the four seasons of the year, and at a fix interval of 15 min. We carried out a factorial analysis of variance (ANOVA, [35]) with FSR as the dependent variable and sex, season and their interaction as independent factors.

## Results

### Stationary assessment

All five collars tested simultaneously at the survey mark to identify faults and consistency collected 100% of locations (FSR = 1). The LE_RMS_ for each collar ranged between 8.7 and 13.6 m. Analysis of variance revealed no significant differences in performance with regard to µLE among the five collars tested calculated from the log transformed (i.e., to meet a normal distribution of data) LE values (F_4,480_ = 1.689, p = 0.151).

Most of the acquired locations from the 60 field sites were fixed utilizing 3 (2-D) or 4 satellites (3-D) (72%) and with HDOP values between 2 and 4 (58%). As expected, LE_RMS_ of 2-D fixes was higher (53.4 m) than for 3-D fixes (22.9 m). However, for the dataset free of outliers (i.e., <3×Stdv), LE_RMS_ of 2-D fixes decreased significantly to 36 m, while LE_RMS_ of 3-D locations was 18.9 m, close to the value obtained for the full dataset. This indicated that most of the outliers occur within 2-D locations, although 78.7% of the fixes accounted for a LE of <30 m.

**Figure 1:**
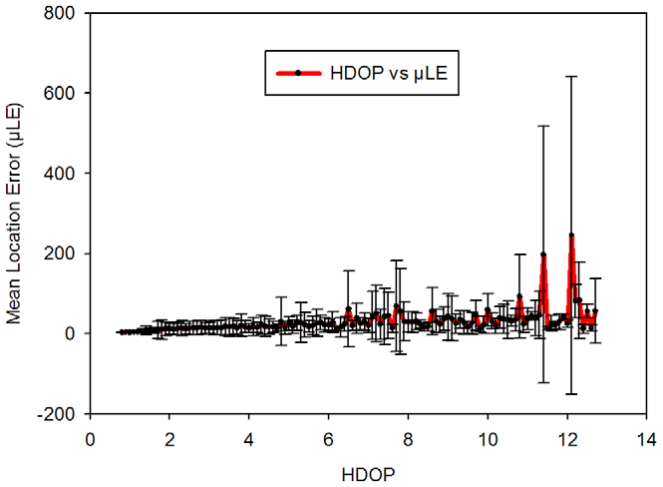
Mean location error (µLE) and standard deviations according to Horizontal Dilution of Precision (HDOP) values.

**Figure 2:**
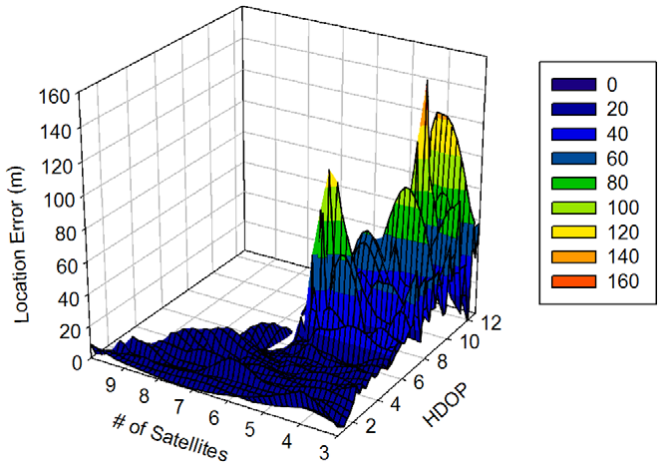
Relationship between Horizontal Dilution of Precision (HDOP), the number of satellites, and the location error (LE).

The calculated µLE per HDOP values remained low and mostly constant for low HDOP up to ca. 4.8 (Figure 1). Between 6.5 and 10 HDOP, µLE fluctuated due to an increase in the proportion of more inaccurate locations, these reaching maximum values for HDOP >10, whereby large outlier values occurred. Locations fixed with more than five satellites were less frequent but typically exhibited a lower HDOP and LE (Figure 2). As expected LE values increased (less accuracy) for positions collected using 3 and 4 satellites and an HDOP varying between 6 and 12. However, LE was often relatively low even for locations fixed with 3–4 satellites. The large variation of µLE for each HDOP value, and standard deviations beyond HDOP of ca. 4.8 (Figure 1) indicates that the proportion of fixes having a low accuracy increases as HDOP increases, but also interspersed with fixes of accuracy similar to the observed for HDOP lower than 4.8 (Figure 1 and 2). Linear regression analysis of logarithm transformed LE (i.e., to meet a normal distribution of data) and HDOP confirmed that an increase in HDOP values was associated with an increase in LE (coefficient = 0.16, SE = 0.004, p<0.0001), although HDOP as an indicator of satellite geometry in the sky explained only 18% of the variation in LE (R^2^ = 0.18). HDOP screening simulations confirmed, as expected, that accuracy increased (LE_RMS_ decreased) with the removal of fixes with HDOP higher than a specific cut-off value. For instance, LE_RMS_ was reduced by nearly 4 m by removing values of HDOP>7 (Table 1). However, improvements of LE_RMS_ occurred at the cost of removing between 40 and 60% of fixes that included between 43.8 and 61% of data with LE<30 m (Table 1). HDOP>12 was considered the most suitable filtering value with removal of locations with the largest LE insuring the maximum number of accurate positions (Table 1).

**Table 1:** Root mean square location error (LE_RMS_) in three percentile ranges after applying HDOP filters on positional data collected at survey mark and stationary habitat sites.

	HDOP filtering thresholds						
Percentile retained	<7 LE_RMS_ (m)	<8 LE_RMS_ (m)	<9 LE_RMS_ (m)	<10 LE_RMS_	<11 LE_RMS_	<12 LE_RMS_	<13 LE_RMS_
				(m)	(m)	(m)	(m)
100%	4.34	4.86	5.33	5.76	6.17	6.53	38.02
95%	4.16	4.65	5.08	5.48	5.84	6.16	15.91
50%	2.66	2.9	3.1	3.28	3.44	3.55	5.27
% Data removed	61	55	50	46	42	40	-

Percentile removed							
100%	89.93	93.39	101.02	109.15	116.9	108.12	
95%	42.61	43.8	47.23	50.58	52.89	55.34	
50%	13.3	14.38	15.41	17.11	18.27	18.94	
% removed LE<30 m	56.1	53.3	50	46.3	44.5	43.8	

The overall percentage of removed data as well as the percentage of this removed data with LE <30 m are given to assess both the amount of positions to be discarded to reduce the average LE, and the loss of suitable data (assumed as LE <30 m) removed by the filtering.

Analysis of the data obtained from the field sites showed that for the visibility category ‘high sky availability’, FSR values were close to 100% for all habitats except native forest (i.e., 37–51%) (Table 2). We discarded from the FSR analysis one ‘native forest’ site because the collar ceased to collect positions probably due to a technical, battery or activation fault. However, we used the LE values for the 5 fixes successfully collected. LE_RMS_ values between habitats ranged from 12 to 47 m and exhibited an increasing trend (i.e., decrease in location accuracy) from ‘no vegetation’ to forested habitats (Table 2). The highest LE_RMS_ was observed for ‘mature pine forest’ (47 m) which is characterized by dense stands. The understorey in native forest resulted in a decrease in FSR (from 51 to 37%) but had no apparent impact on LE_RMS_ (ca. 36–37 m). For most of the habitats (as tested for various sky availability configurations) LE_RMS_ tended to increase with decreasing sky availability, except for ‘native forest’ under ‘low sky availability’ whereby LE_RMS_ actually decreased from 131 m to 78 m. Overall, location accuracy remained within 30 m outside forested habitats. Highest sky availability consistently resulted in a higher FSR compared to lower sky availability, except for ‘native forest’ whereby the FSR was lower with ‘high sky availability’ followed by low and medium categories (Table 2). The effect of sky availability for vegetation categories other than forest and shrubs (i.e., ‘no vegetation’, ‘low vegetation’ and ‘tussockland’) appears negligible with only limited decrease in FSR (i.e., 0.4%) and slight variations in LE_RMS_. µLE of some vegetation/topography categories had large standard deviations and median values much lower than the mean indicating a skewed distribution of data (Table 2) due to the presence of some fixes with high LE. Filtering of fixes with LE<3×Stdv of population per site showed that during a 24 h-observation period, one or two fixes exhibited large abnormal LE. Removal of these outliers led to improvement in the LE_RMS_ values per habitat type by several meters (Table 2).

**Table 2:** Comparison between fix success rate (FSR) ± standard deviation and root mean square of location errors (LE_RMS_), mean location errors (µLE) ± standard deviation and median (µ_1/2_LE) obtained from analysis of data collected at stationary tests (N = 60) under different habitats, vegetation configuration and sky availability.

Habitat	High sky availability				Medium sky availability				Low sky availability				Total				Outliers	
	FSR (%)	LE_RMS_ (m)	µLE (m)	µ_1/2_LE (m)	FSR (%)	LE_RMS_ (m)	µLE (m)	µ_1/2_LE (m)	FSR (%)	LE_RMS_ (m)	µLE (m)	µ_1/2_LE (m)	FSR (%)	LE_RMS_ (m)	µLE (m)	µ_1/2_LE (m)	Mean number outliers	LE_RMS_ (m)
No Vegetation	100	14.6	9.2±11.3	6.1	100	26.6	13.5±23.4	8.4	99.6±0.5	25.8	19.2±17.2	15.6	99.8±0.1	23	14±18.4	8.9	1.3±0.8	17.9
Low Vegetation	100	12.1	7.9±9.2	5									100	12.1	7.9±9.2	5	1.7± 2	9.7
Tussocks (50–75%)	100	17.6	8.8±15.2	5.9									100	17.6	8.8±15.2	5.9	1.6±0.6	10.2
Tussocks (>80%)	97.6±2.0	12.3	9.1±8.2	6.8	100	19.7	12.4±15.4	8.1	99.6±0.5	23.7	14.9±18.4	9.4	98.7±2	17.7	11.4±13.6	7.7	1.8±0.7	14.2
Shrubs (50–75%)	99.8±0.4	19.2	11.7±15.2	7.4									99.8±1	19.2	11.7±15.2	7.4	1.8±1.1	13.7
Shrubs (>80%)	96.6±4.0	16.3	11.7±11.4	8.5	99.6±0.5	21.7	12.1±18.0	7.8	94.6±5	29.3	19.2±22.1	11.9	97±4	21.5	14.3±18.3	9.1	2.2±0.6	15.1
Mature pine forest	98.3±0.5	47.3	31.0±35.8	22.4									98.3±0.5	47.3	31.0±35.8	22.4	2.0±0.0	33.3
Native forest	51 ± 2.0	36	30.7±18.9	26.7	87.3±20	131.1	67.9±112.4	32.6	74.3±12	78	47.2±62.2	28.1	70.8±20	92.3	51.7±82.5	29	1.6±1.1	70.2
Native forest with understorey	37 ± 17	37.6	33.4±17.8	29.2									37±12.7	37.6	33.4±17.8	29.2	0.3±0.5	35.3
**Total**													89±21	39.5	17.9±35.3	9.2	1.6±0.9	27.6

Outliers correspond to fixes with location error (LE)>3 standard deviations from the mean location error of all fixes in the same habitat (i.e., without regard to the visibility category). The last two columns report on the mean number of outliers ± standard deviation across each visibility, and LE_RMS_ values calculated from all fixes in the same habitat after removal of outlier values.

Model selection for FSR based on the *Q*AIC_c_ gave support mostly for the two most plausible models [43] (ΔQAIC<2) for FSR (Table 3). The top-ranked model included ‘tree cover’ and ‘shrub cover’, while the second-ranked model contained ‘low vegetation cover’ in addition to the aforementioned variables. Given the model-averaged coefficients for the predictors, i.e., ‘tree cover’ (coefficient = −4.59, SE = 1.93), ‘shrub cover’ (coefficient = −2.62, SE = 1.43) and ‘low vegetation cover’ (coefficient = −0.11, SE = 1.21), we inferred that FSR decreases with increasing tree and shrub cover. The predominance of these vegetation components represents the main vegetation classes in native forest with understorey.

**Table 3:** Models explaining the fix success rate (FSR) of lightweight-GPS collars tested in stationary sites (N = 59) under different habitats, vegetation configuration and sky availability.

Rank	Model description	K	*Q*AIC_c_	Δ*Q*AIC_c_	*ω*
1	Tree cover + Shrub cover	4	99.32	0	0.57
2	Tree cover + Shrub cover + Low vegetation cover	5	101.19	1.87	0.22
3	Tree cover + Shrub cover + Low vegetation cover + Sky availability	6	101.37	2.04	0.2

All candidate models represented alternative hypotheses expressed as logistic models. The response variable registered the percentage of successful fixes. Models are ranked from the most explanatory model after Akaike Information Criterion (AIC) diagnosis for small and dispersed sample size (*Q*AIC_c_); K indicates the number of parameters; Δ*Q*AIC_c_ the change in *Q*AIC_c_ and *ω* values of weighted analysis.

Model selection for LE based on AIC revealed three most plausible models (ΔAIC<2). The top-ranked model included ‘tree cover’, ‘sky availability’, ‘number of satellites’ and HDOP. The other two models incorporated the top-ranked model variables along with ‘shrub cover’ and ‘low vegetation cover’ (second-ranked model), or ‘low vegetation cover’ (third-ranked model) (Table 4). We model-averaged the fixed effects for ‘tree cover’ (coefficient = 1.34, SE = 0.10), ‘shrub cover’ (coefficient = 0.04, SE = 0.02), ‘low vegetation cover’ (coefficient = −0.02, SE = 0.06), ‘sky availability’ (coefficient = −0.001, SE = 0.006 SE), ‘number of satellites’ (coefficient = −0.06, SE = 0.01), and HDOP (coefficient = 0.1, SE = 0.007). The ‘tree cover’ had the strongest effect on LE with LE increasing as tree cover increases.

**Table 4:** Models explaining location error (LE) of lightweight-GPS collars tested in stationary sites (N = 60) under different habitats, vegetation configuration and sky availability.

Rank	Model description	K	LL	AIC	ΔAIC	*ω*
1	Tree cover + Sky availability + #Satellites + HDOP	7	6044.1	12102.2	0	0.42
2	Tree cover + Sky availability + #Satellites + HDOP + Shrub cover + Low vegetation cover	9	6042.8	12103.5	1.27	0.22
3	Tree cover + Sky availability + #Satellites + HDOP + Low vegetation cover	8	6044.1	12104.2	1.99	0.16

Candidate models represented alternative hypotheses of Linear Mixed Models (LMMs) with site as the random effect and log(LE) as the response variable for each fix. Models are ranked from the most explanatory model after Akaike Information Criterion (AIC); K indicates the number of parameters; ΔAIC the change in AIC and *ω* values of weighted analysis.

### Motion and behavioural assessment

The length of the five routes travelled varied from 1.8 to 9.7 km with durations of between 48 and 253 min (Table 5). Four of the routes yielded a high FSR value ranging from 86% to 100%. The fifth track had the lowest FSR (79%), was the shortest in both space and time, and followed numerous direction changes in an area of varied vegetation, resulting in few extended longitudinal segments. Highest FSR values were obtained for the two longest itineraries where a few long straight segments were covered through varied vegetation. LE_RMS_ showed similar tendency to that of FSR. The highest LE_RMS_ value was 50.2 m and corresponded to the shortest itinerary. On average FSR values were found to be ca. 10% lower (90%±3%) and location accuracies ca. 15 m less accurate (29.8 m) than the static tests carried out in ‘high sky availability’ and similar habitats (i.e., ‘low vegetation’, ‘tussockland’ and ‘shrubland’) (Table 2 and 5).

**Table 5:** Fix success rate (FSR) and root mean square of location errors (LE_RMS_) results for the cat itineraries simulated in the field.

Itinerary	N	Distance (m)	Time (min)	FSR	LE_RMS_ (m)
1	227	9725	253	89%	31.9
2	181	8765	190	96%	25.2
3	38	1820	48	79%	50.2
4	50	1984	58	86%	23.8
5	60	2406	60	100%	14.1
Total	556	24700	609	90%±3%	29.8

Of the 19 female cats (N = 19) equipped with the lightweight GPS-collars, 5 were tracked in summer and autumn, 6 in winter and 3 in spring. FSR values for females ranged from 36% to 86% (mean = 63%±3%), steadily decreased from summer to winter, and increased again to autumn levels in spring (Figure S3). We tracked 2 males in summer, 9 in autumn, 9 in winter and 4 in spring (N = 24) yielding FSR values ranging from 37% to 93% (mean = 64%±3%). Values were similar in summer and autumn, increased in winter and were at their maximum in spring (Figure S3). However, the male sample in summer included only two individuals, one with a low and one with a high FSR. Results from factorial ANOVA revealed no significant differences between sex, seasons and the interaction of sex and seasons (*F*_3,35_ = 1.47, p = 0.23).

## Discussion

We first assessed the consistency in the performance of the five units used in this research. Testing the collars in identical conditions (i.e., placed simultaneously over a survey mark) is necessary to identify manufacturing malfunction or potential electronic discrepancies between units. We observed similar performance for all collars in both LE and FSR and no malfunction in agreement with the results from Blackie [14] who also utilized Sirtrack collars.

We found that the lightweight collars deployed at the stationary sites operated normally. Of the 60 sites, the FSR value for a single native forest site was omitted from the analysis due to collar failure. Average stationary FSR was 89% (considering all habitat and sky availability categories), similar to the results obtained by Dennis et al. [13] with lightweight GPS-collars of the same brand. All FSR values except those from native forests were over the 94.8% reported by similar tests carried out with bigger collars in large mammals [29]. The dense vegetation characterising New Zealand native forest was associated with the largest loss of fixes, especially in sites with understorey (only 37% fixes were successfully acquired); this concurs with model selection indicating tree and shrub densities as the main factor affecting FSR.

The obtained LE_RMS_ values in the stationary tests are comparable to the accuracies previously reported in wildlife telemetry of ±30 m [3], except for the forest categories ranging from 37 to 92 m across the different sky categories. We found that LE_RMS_ values were similar in control sites without vegetation and in sites with ‘low vegetation’ and ‘tussockland’. Tussockland is a common habitat in New Zealand, often composed of dense associations of large tussock species (±80 cm tall) such as tall *Chionochloa* spp. Our results reveal the negligible impact of low vegetation and tussock categories in the performance of lightweight GPS-collars, thus medium-to-small mammals tracked in areas with this vegetation are expected to derive suitable location datasets with limited error.

LE_RMS_ decreased for all habitat categories to between ca. 10 m to 70 m after filtering outliers, which indicates the importance of applying preliminary data filtering to remove these locations from the raw dataset. Highest LE_RMS_ values still were associated with forests after filtering. Modelling of LE identified that once a position is fixed, its accuracy depends on a combination of vegetation structure primarily associated with forest, and secondarily topography (sky availability), number of satellites, and their geometry in the sky (HDOP). Researchers aiming to track medium-to-small mammals in forest environments need to consider limitations in the technology during the formulation of hypotheses and experimental design, and for the analysis of collected data, which may require application of corrections for both FSR and LE [3].

HDOP values associated with successful fixes explained 21% of the variation of LE, thus confirming that a poor geometry of satellites contributes to increased LE, although it is not the only determinant. D'Eon and Delparte [21] identified the inconsistency between data with high DOP values and low LE, making it difficult to derive efficient data screening based solely on DOP values. We found similar results with our lightweight GPS-collars and we agree with Lewis et al. [22] that any filtering method based on high HDOP values will yield only limited reduction of GPS errors at the expense of discarding a potentially large number of accurate fixes. In regard to the number of satellites we also agree with D'Eon et al. [44] that filtering of 2-D locations needs to be used cautiously, if at all. Locations fixed with 3 satellites (2-D) have higher LE_RMS_ mainly because of the presence of outliers, although many accurate fixes also occur in 2-D locations. Moreover, as it happened in our research, the proportion of 2-D fixes can be high and their removal can considerably reduce the dataset and result in substantial loss of information. Alternative methods of LE screening rely on movement characteristics of the species, for instance, filtering points implying unrealistic speed, turning angles and/or directional bias [45], or the use of non-linear state-space models incorporating an error term [46]. However, these methods generally require high temporal frequency data or for the latest approach, mathematical formulations that may not be accessible to most biologists. Hence, we recommend DOP filtering only if reduction in dataset size can be accommodated. Considerations based on research objectives and analysis techniques are required to choose the most suitable screening method. Projects investigating animal movement patterns should preserve as many positions as possible and apply available corrections for accurate estimations. However, projects focused on space use without an underlying movement model and relying on abundant positional data can accommodate the removal of a certain proportion of locations.

On average, motion tests produced 10% fewer successful fixes compared to the static tests (considering the same habitat configuration, i.e., ‘high sky availability’ and ‘no vegetation, ‘low vegetation’, ‘tussockland’ and ‘shrubland’), whereas location accuracies where found to be approximately 15 m less accurate. As with the stationary tests, LE values were within ±30 m except for one itinerary with ±50 m, and which also had a relatively low FSR (79%). This test was the shortest in time and distance, and was carried out in the same types of habitat as the other itineraries. However, this short time coincided with a period of suboptimal number and geometry of satellites in the sky that might explain the lower performance. Hence, LE comparisons between stationary and motion tests need to be made with caution. Indeed, the Garmin GPS is not as accurate as the Leica GS20 with differential correction (sub-meter error for the Leica and ±4.5 m for the Garmin assessed over a geodetic point). In addition, the distance between the operator (carrying the reference GPS) and the collar was ±2 m, and the time difference between the corresponding collar and reference fixes was up to 2 seconds (corresponding to about 1.3 m at a speed of 2 km/h). Ultimately, simulated tracks were more likely to be between 10 and 20 m less accurate than the static tests.

Previous motion tests utilized GPS-collars on dogs rambling under forests with the operator following at a certain distance and carrying the reference GPS [25], or attached to cars driving along roads under forest [27]. These experiments also revealed that collar performance in motion tests was lower than in stationary, coinciding with the conclusions of Edenius [47] and Biggs et al. [31] reporting a reduction of FSR for moving collars on moose and elk, respectively. However, accurate motion tests are difficult to perform, as the reference GPS also must acquire accurate positions to reduce biased LE calculations; this objective can be difficult to achieve under forest or dense shrublands. The use of micro-topographical and habitat features is also difficult to simulate (e.g., movement under shrub clumps). Moreover, the data analysis needs to account for synchronized time differences between the reference GPS and the GPS-collar, and the distance between them. However, results derived from motion tests can approximate the performance of GPS-collars to more realistic scenarios where error factors (e.g., multipath, vegetation, topography) variably occur in comparison with controlled stationary tests.

Stationary and motion FSR values were higher than those obtained from deployment on wild animals. Our results for the feral cats tracked yielded a FSR of 64%, similar to the 64.8% (range 24.7 to 74.0%) found by Blackie [14] but lower than the 87.6% reported by Dennis et al. [13] using Sirtrack collars on brushtail possums. Our results are also similar to the average FSR values reported for other medium to small mammal species such as one ocelot with 61% FSR [12] but higher than the 20% reported by Sprague et al. [11] on one Japanese macaque under forest. This gap between stationary tests and real deployment on animals indicates that stationary results should be used with caution and highlights the importance of animal behaviour on the success of fixes.

In all of the tests we used fix rates (15 min and 1 min) that imply a ‘hot start’ condition during location acquisition (see [48]). A hot start means the presence in the GPS memory of a current almanac, time, location and ephemeris information for each satellite in sight derived from the last location. Hence, in hot start conditions, the more frequently the locations are collected, the more accurate they are [48]. Further research is required to identify the effect of a ‘warm start’ (using current almanac, time and position, but without a current ephemeris) required for intervals >2–4 h between locations due to, for instance, when an animal is out of sight of satellites for a few consecutive fixes.

Behavioural influences are an important source of error for lightweight collars deployed on small mammals, although FSR values are within the range of those reported for heavier collars placed on larger species. D'Eon [24] identified animal behaviour as the main source of data loss (low FSR) dismissing stationary tests as a suitable method to account for most missing data. However, a stationary test is an appropriate technique to isolate vegetation and topographical effects from behavioural factors. Behavioural factors particular to the focal study species need to be carefully considered by researchers in spite of the expectations created by technological developments. Smaller mammals can tend to use micro-habitats with partially or fully blocked sky (e.g., cavities or holes). Hence, there is a need to define the appropriate tracking schedules (avoiding probable periods of use of these micro-habitats, such as day/night, hibernating), the accuracy required, and the degree to which these limitations and adjustments may compromise research objectives when considering the deployment of lightweight GPS. Another consideration based on behaviour is the movement range of the study species, whereby robustness of habitat use models in short-ranging species can be compromised by location errors that could be acceptable in large and wide-ranging species. In spite of the many opportunities that GPS-tracking offers wildlife research [1], the application of this tool should be conditioned by its technological limitations and how these may compromise research objectives.

Improvements in GPS miniaturization will expand the range of mammal species that can be tracked, considering only device size and weight limitations. However, other technological developments are required to improve signal reception under conditions of suboptimal sky availability. Further miniaturization of receivers and other technological improvements will continue to reduce the size and weight of devices, unit power demands, and the efficiency of antennae. These advances will result in more efficient receivers which can better operate under challenging conditions such as restricted sky availability or dense forest canopies, on smaller species, and for longer periods. Performance tests will continue to be required to assess improvements in device performance and associated errors. Researchers should base their research on well-tested GPS devices, although specific tests are recommended to assess the device suitability for specific project objectives, study species and their habitats. Special consideration should be given to species inhabiting forest as location accuracy and fix success are more seriously affected, and further research is required, for instance, to evaluate FSR and LE along a vertical range of arboreal or semi-arboreal mammal species such as brushtail possums.

We have demonstrated the suitability of lightweight GPS-technology to track medium to small mammal species, which extends the range of species that can be tracked, and therefore can assist us in increasing our knowledge of animal ecology. Ongoing advances in GPS tracking could give rise to a future where it might be possible to track any species of any size, using microGPS receivers with a standard performance and a low LE under all environmental configurations or animal behaviours.

## Supporting Information

Figure S1 **Sky obstruction model.** Sky obstruction by surrounding slopes (adapted from Sirguey et al. 2009). It is measured by horizon lines *H(ϕ)* of the point under consideration in all azimuth angles *ϕ* . The sky availability *V_d_* is defined as the ratio between the solid angle subtended by the horizon lines and *2π*.
(TIF)

Figure S2 **Pivoting collar support utilized for mobility test.**
(TIF)

Figure S3 **Fix success rate results of collars deployed on cats.** Comparison of mean fix success rates (FSR) ± standard deviation obtained from male (N = 24) and female (N = 19) feral cats tracked in the Godley and Tasman Valley in the Central South Island, New Zealand.
(TIF)
