# Supplementary figures and images for: Lightweight GPS-Tags, One Giant Leap for Wildlife Tracking? An Assessment Approach
Source: PLoS One. 2011 Dec 7;6(12):e28225. doi: 10.1371/journal.pone.0028225 (PMC3233555; doi:10.1371/journal.pone.0028225)

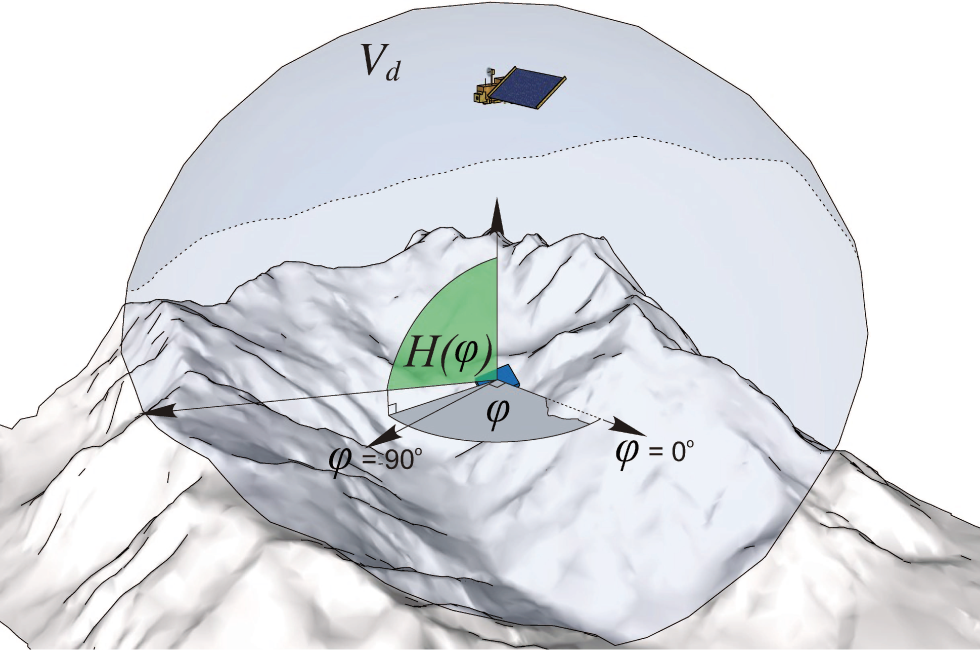

Supplement: Figure S1 — Sky obstruction model. Sky obstruction by surrounding slopes (adapted from Sirguey et al. 2009). It is measured by horizon lines H(ϕ) of the point under consideration in all azimuth angles ϕ . The sky availability Vd is defined as the ratio between the solid angle subtended by the horizon lines and 2π. (TIF) [file pone.0028225.s001.tif]

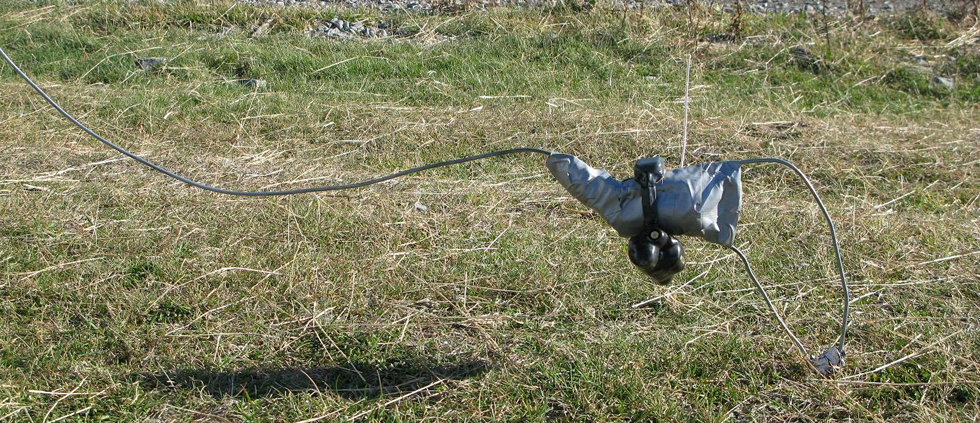

Supplement: Figure S2 — Pivoting collar support utilized for mobility test. (TIF) [file pone.0028225.s002.tif]

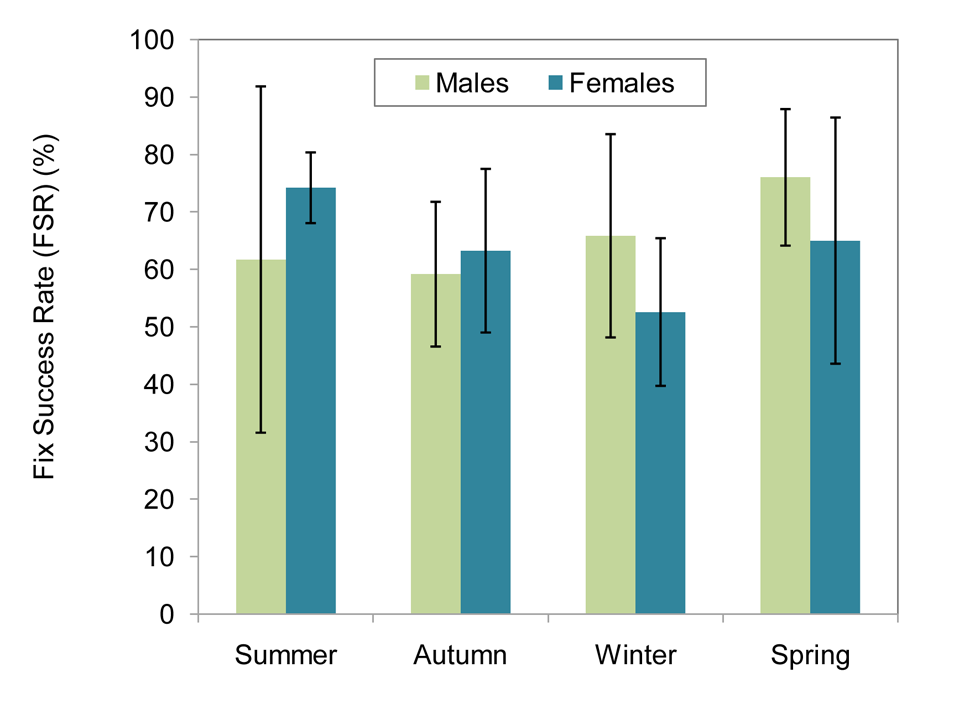

Supplement: Figure S3 — Fix success rate results of collars deployed on cats. Comparison of mean fix success rates (FSR) ± standard deviation obtained from male (N = 24) and female (N = 19) feral cats tracked in the Godley and Tasman Valley in the Central South Island, New Zealand. (TIF) [file pone.0028225.s003.tif]
